# Role of Th22 Cells in Human Viral Diseases

**DOI:** 10.3389/fmed.2021.708140

**Published:** 2021-08-09

**Authors:** Jianguang Gong, Huifang Zhan, Yan Liang, Qiang He, Dawei Cui

**Affiliations:** ^1^Department of Nephrology, Nephrology Center, Zhejiang Provincial People's Hospital, Affiliated People's Hospital, Hangzhou Medical College, Hangzhou, China; ^2^Department of Emergency, Zhejiang University Hospital, Hangzhou, China; ^3^Zhejiang Academy of Medical Sciences, Hangzhou, China; ^4^Department of Blood Transfusion, The First Affiliated Hospital, Zhejiang University School of Medicine, Hangzhou, China

**Keywords:** naive CD4^+^ T cells, Th22 cell, IL-22, virus hepatitis, hand, foot, and mouth disease, HIV disease, COVID-19

## Abstract

Naive CD4^+^ T cells can differentiate into different cell subsets after receiving antigen stimulation, which secrete corresponding characteristic cytokines and thereby exert biological effects in various diseases. Th22 cells, a novel subset of CD4^+^ T cells, are different from Th1, Th2, Th17, and Treg cell subsets, which have been discovered in recent years. They can express CCR4, CCR6, and CCR10 molecules and secrete IL-22, IL-13, and TNF-α. They are not able to secrete IL-17, IL-4, and interferon-γ (IFN-γ). IL-22 is considered as a major effector molecule of Th22 cells whose functions and mechanisms of regulating cell differentiation have been constantly improved. In this review, we provide an overview of the origin, differentiation of Th22 cells. Moreover, we also describe the interrelationships between Th22 cells and Th17, Th1, and Th2 cells. Additionally, the role of Th22 cells were discussed in human diseases with virus infection, which will provide novel insight for the prevention and treatment of viral infection in human.

## Introduction

T lymphocytes are a significant component of the human immune system and can be further classified as CD4^+^ T cells and CD8^+^ T cells. According to functions, surface markers, and secreted effector molecules, CD4^+^ T cells, also known as T helper (Th) cells were mainly divided into Th1, Th2, Th17, T follicular helper (Tfh) cells, regulatory T (Treg) cells, and other Th cell subsets (1, 2). The different subsets of Th cells subsets play important roles in the development and progression of human autoimmune diseases, infections, and tumors (3, 4). During research to further understand Th cell subsets, new subgroups have been gradually discovered. In the early phases, studies have demonstrated that both Th17 and Th1 could secrete IL-22 (5, 6). Further studies on IL-22 found a group of special cell subsets that can secrete IL-22 and IL-13 instead of IL-17, IL-4, and IFN-γ. These subsets were later confirmed to be CD4^+^ T cell subsets and independent of Th1, Th2 and Th17 cells. This subset of cells was named Th22 (7). Th22 cells can express CCR4, CCR6, CCR10 and several fibroblast growth factors (FGFs) molecules. They also participate in the homeostatic regulation of skin and pathological processes (7), promote angiogenesis, and accelerate wound healing (8). IL-6 and TNF-α can induce the differentiation of naive CD4^+^ T cells into Th22 cells, while TGF-β was found to inhibit differentiation (7).

Viral infectious diseases, such as Acquired Immune Deficiency Syndrome (AIDS), viral hepatitis and so on, seriously threaten human health, and become one of the problems that people need to solve. Recent studies have shown Th22 cells may be involved in regulating the pathological processes of many viral infectious diseases (9–11). The traditional concept of simple division of Th cells is continuously being renovated. This speaks to the diversity of T cell functional subsets and the complexity of immune regulatory functions. As a novel Th cell subset, Th22 cells further expand the understanding of immune regulation (8, 12). The differentiation and role of Th22 cells and their relationships with other T cell subsets are extremely important for recognizing T cell immune response. These findings will also help with understanding the pathogenesis of diseases and exploring more effective targets of disease intervention. This review will focus on the differentiation and regulation of Th22 cells and discuss the research progress for the role of Th22 cells in several common human viral diseases.

## The Origin of TH22 Cells

The origin of Th22 cells was derived from a study on IL-22. In 2000, Dunamtier et al. used IL-9 to stimulate mice lymphoma cells. They found that these cells expressed a cytokine closely resembling the secondary structure of IL-10. They named this cytokine Interleukin-10-related T cell-derived inducible factor (IL-TIF) (13). Also in 2000, Gurney et al. identified a new sequence from T cells isolated from humans with 23% of the encoded amino acids being homologous to IL-10, and 87% were similar to IL-TIF (14). This sequence was designated as IL-22. Initially, IL-22 was considered to be a cytokine associated with T helper type 1 cells (Th1) (5, 15). It was later found to be closely related to the expression of IL-17 by IL-17-producing T helper cells (Th17 cells) (6, 16). IL-22 can also be derived from natural killer T cells (17) and lymphoid tissue–inducer cells (LTi cells) (18). In mice, IL-22 is mainly produced by Th17 cells. Retinoid-related orphan receptor-γt (ROR-γt), known as RORC in humans, is a transcription factor that controls the generation of Th17 cells. Retroviruses were used to transfect ROR-γt into T cells of mice, which were endowed with the ability to express IL-17 and IL-22 (19, 20). Human T cells transfected with RORC cannot induce IL-22 expression (21). Another transcriptional regulator of Th17 cells is Aryl hydrocarbon receptor (AHR). AHR can also promote the expression of IL-17 and IL-22 (22).

In 2009, Sara Trifari et al. identified a group of CD4^+^ memory T cells with the phenotype of CCR4^+^CCR6^+^CCR10^+^ in the blood of healthy adults. These T cells produced IL-22 and IL-13, but did not secrete IL-17 and IFN–γ. The expression of IL-22 could be promoted by upregulation of AHR or the transcription factor RORC (23). In 2009, Duhen et al. classified CD4^+^CD45RA^−^CD25^−^ memory T cells isolated from peripheral blood of healthy individuals (7). Cells were grouped according to whether they expressed CCR6, and the expressions of IL-17, IL-22, and IFN-γ were analyzed. After polyclonal stimulation, both intracellular and cultured supernatants showed expression of IL-17 and IL-22 was completely restricted to CCR6^+^ subset cells, while IFN-γ was expressed in both CCR6^+^ and CCR6^−^ subsets. Based on the biological function of IL-22, the researchers hypothesized that IL-22- producing cells might have the characteristics of skin-homing T cells, so T cells were separated into four subsets according to the expression of CCR6, CCR4, and CCR10. Further analysis showed that CCR10^+^CCR6^+^CCR4^+^ subset cells only expressed IL-22. Neither IL-17 nor IFN-γ was expressed after stimulation with anti-CD3 and anti-CD28. Researchers confirmed the independence of Th22 cells through the following experiments (8). They placed Th22 cell clones derived from patients with psoriasis in an inducible environment of Th1 cells, Th2 cells, Th17 cells, and Treg cells. They found that these cells maintained the ability to secrete IL-22 and did not secrete the characteristic cytokines of other T cell subsets. Thus, it can be determined that Th22 cells are an independent and stable lineage. In 2017, Plank et al. carried out the whole gene chip to analyze the mRNA transcription profile of Th22 and Th17 cells (24). This research further confirmed the differences between Th22 and Th17 cells.

## Molecular Characteristics and Differentiation of TH22 Cells

**Figure 1:**
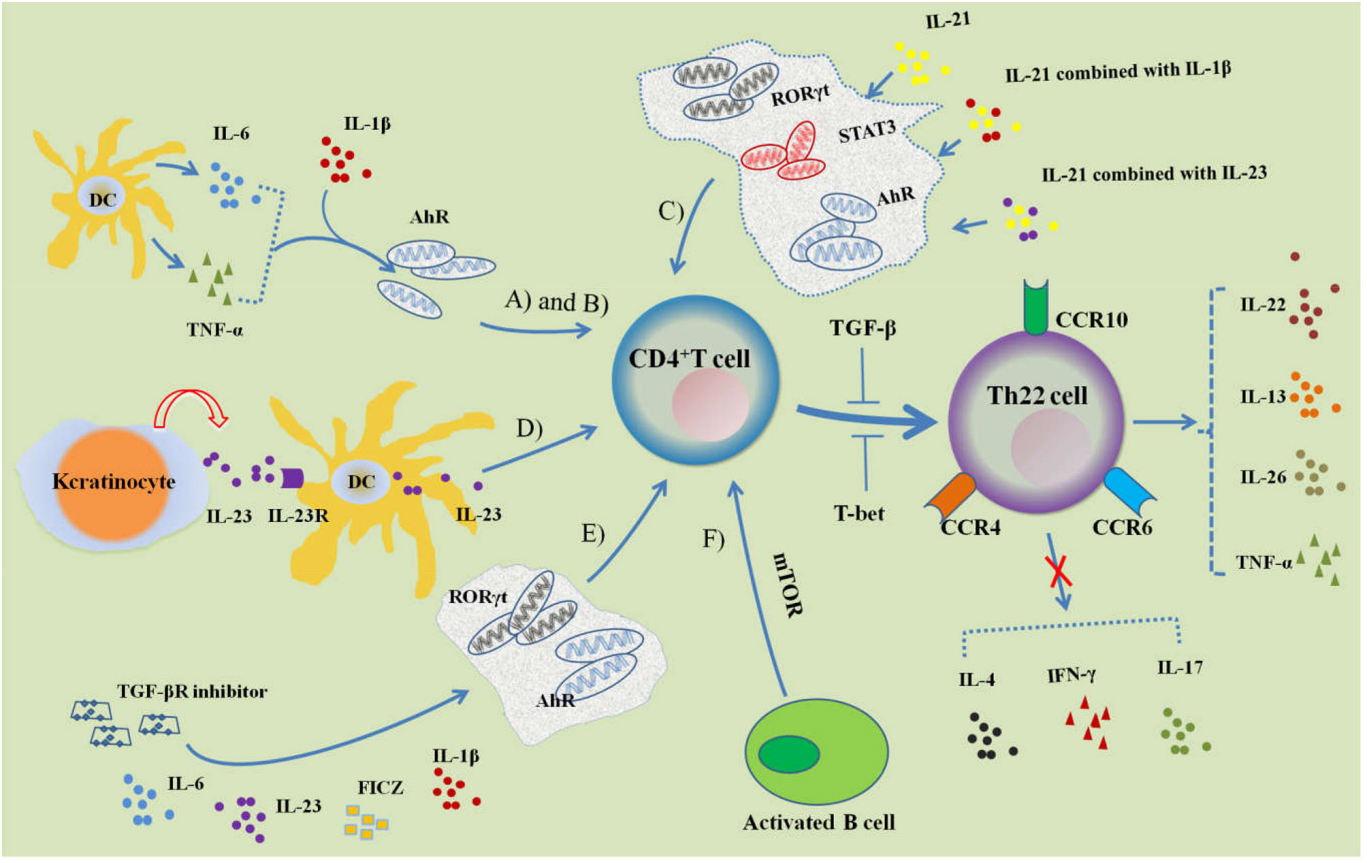
Regulation of Th22 cell differentiation. **(A)** IL-6 and TNF-α induce naive CD4^+^ T cells to differentiate into Th22 cells characterized with CCR4, CCR6, CCR10 and IL-22 expression, which can be promoted by IL-1 β. AhR is involved in the regulation of differentiation. **(B)** Activated DCs can secrete IL-6 and TNF-α to induce Th22 cell differentiation. **(C)** IL-21, IL-21 combined with IL-23 or IL-1β can induce Th22 cell differentiation and IL-22 expression, while the transcription factors STAT3, RORγt, and AhR participate in its regulation. **(D)** Keratinocytes are stimulated by endogenous TLR4 ligands and secrete IL-23, which combines with IL-23R of DCs; activated DCs further secrete IL-23 and induce CD4^+^ T cells to differentiate into Th22 cells. **(E)** Combined with IL-6, IL-23, IL-1β, FICZ and TGF-βR inhibitors can promote the differentiation of CD4^+^ T cells into Th22 cells. **(F)** Activated B cells can induce Th22 cell differentiation by activating mTOR signaling. Both TGF-β and T-bet can inhibit the differentiation of Th22 cells.

Th22 cells are a novel subset of CD4^+^ Th cells that are distinct from Th1, Th2, and Th17 cells (Figure 1). Th22 cells are able to secrete IL-22, IL-26, IL-13, TNF-α, and granzyme B (24). They are not able to produce INF-γ, IL-4, or IL-17, and IL-22 is its main effector molecule. Th22 clones begin to release IL-22 at the 6th h and reach a peak at the 12th h. The expression of IL-22 can be maintained at this level for 48 hours. Th22 cells have a CD3^+^CD4^+^ phenotype. In addition, they also express the skin chemokine receptors CCR4, CCR6, and CCR10. CD8 and NK cell markers CD56, NKp44, and NKp46 were found to be negative (8). Human skin typically expresses abundant levels of chemokines. This explains why there are relatively more Th22 cells found in the skin and fewer found in circulation throughout the body.

The differentiation of Th22 cells is regulated by many factors, mainly involving into the cytokine, cellular membrane molecules, and transcription factors. Duhen et al. demonstrated that IL-6 and TNF-α together jointly induced Th22 cell differentiation (7). For this process, IL-6 was crucial for Th22 cell differentiation and stimulation with IL-6 alone resulted in a substantial differentiation of naive CD4^+^ T cells into Th22 cells. This suggests that IL-6 may be a priming factor for Th22 cell differentiation. When IL-6 and TNF were used in combination, researchers found that the proportion of Th22 cells was higher than when IL-6 was used alone. This suggests that TNF may play a role in promoting Th22 cell differentiation. High doses of TGF-β exerted an inhibitory effect on the differentiation of Th22 cells. Plank et al. optimized the condition of Th22 cell differentiation (24). For intervention, they first combined with four factors: IL-6, IL-23, IL-1β, and 6-formylindolo [3,2-*b*] carbazole (FICZ). This study found that IL-17A was still secreted. In order to inhibit IL-17A secretion, researchers added a TGF-βR inhibitor (galunisertib), which effectively inhibited IL-17A production without affecting the secretion of IL-22. IL-13 and granzyme B levels were also significantly increased under this culture condition. IL-21 and IL-23 can also induce the differentiation of Th22 cells. Yeste et al. found that either IL-21 alone, IL-21 combined with IL-23 or IL-1β could induce Th22 cell differentiation and IL-22 expression (25). The transcription factors signal transducer and activator of transcription 3 (STAT3), RORγt, and AhR were involved in their regulation. The endogenous TLR4 ligand stimulates keratinocytes to secrete IL-23 and binds to the IL-23 receptor of skin dendritic cells. This activates the secretion of endogenous IL-23, induces the differentiation of naive T cells into Th22 cells, and releases IL−22 (26). Th22 cells are different from other Th cell subsets for characteristic molecules, and the differentiation of Th22 cells are associated with the important cytokines and transcript factors.

Duhen et al. used conventional DCs (cDCs) and plasmacytoid-like DCs (pDCs) to stimulate naive CD4^+^ T cells (7). This research revealed that pDCs had a stronger induction effect on Th22 cells than cDCs. The addition of mature pDCs to the culture medium of stimulated differentiated cDCs promoted the differentiation of Th22 cells, while adding mature cDCs to the culture medium of stimulated differentiated pDCs and inhibited the differentiation of Th22 cells. These results indicate that mature pDCs may indirectly drive the differentiation of naive CD4^+^ T cells into Th22 cells. Further studies found that both cDCs and pDCs released high concentrations of TNF-α and IL-6 after activation, without secreting IL-12, IL-23, and IL-1β. In addition, cDCs were able to produce a small amount of IL-10. This experiment showed that blocking TNF and IL-6 inhibited 70% of Th22 differentiation. These results suggest that DCs may promote Th22 differentiation in both direct and indirect ways. Fujita et al. isolated Langerhans cells (LCs) that were HLA-DR^+^ and CD207^+^ (27). They also isolated dermal DCs (HLA-DR^hi^CD11c^+^BDCA-1^+^ cells) from the epidermis and dermis of normal people. This could induce peripheral blood T cells and naive CD4^+^ T cells to differentiate into Th22 cells, prompting the idea that cutaneous resident DCs may regulate the differentiation of T cells into Th22 and enter the skin. Foreign antigens such as microbes can activate DCs, promote the production of cytokines, and then induce the differentiation of Th22 cells. Díaz-Zúñiga et al. showed that DCs and naive CD4^+^ T cells isolated from the peripheral blood of healthy people were stimulated by different aggregatibacter actinomycetemcomitans serotypes (28). They found that the levels of TNF-α and IL-6 were significantly increased as well as the expressions of IL-22 and AhR, which might initiate the polarization of Th22 cells.

*In vitro*, activated B cells and naive T cells were co-cultured under Th17 cell culture conditions. This revealed that activated B cells could significantly inhibit the production of IL-17 and the expression of RORγt. In contrast, they could stimulate Th22 differentiation and IL-22 production. Further studies *in vivo* demonstrated that treatment with injection of activated B cells in the MRL/lpr lupus mice models reduced anti-dsDNA antibody and protein levels in urine. This also suppressed Th17 cell differentiation and enhanced Th22 cell differentiation (29). The aryl hydrocarbon receptor (AhR) is an endogenous ligand nuclear transcription factor. Activated AhR can significantly promote the differentiation of naive CD4^+^ T cells into Th22 cells (23). Runt-related transcription factor 3 (RUNX3) is a Runt-domain family transcription factor. Studies have shown that RUNX3 is involved in the differentiation of Th22 cells in patients with psoriasis (30). The number of Th22 cells decreased significantly after RUNX3 levels of CD4^+^ T cells from psoriasis patients were restricted by RNA interference. Plank *et al*. isolated naive CD4^+^ T cells from Rorc (γt) knockout mice and found a partial reduction in IL-22 expression under Th22 culture conditions, and naive CD4^+^ T cells that came from Tbx21 knockouts and were cultured in Th22 conditions exhibited a more than two-fold increase in IL-22 expression (24). It is believed that RORγt is partly involved in the differentiation of Th22 cells, while T-bet inhibits the differentiation of Th22 cells. These findings indicated that Th22 cell differentiation could be initiated by innate immune cells including DCs, LCs and B cells that expressed cytokines and membrane molecules.

A recent study indicated that when CD4^+^ T cells isolated from peripheral blood of patients with coronary heart disease were transfected with miR-31 mimic, the differentiation of Th22 cells and the expression of transcription-related factor AHR were significantly promoted (31). This also remarkably accelerated IL-22 secretion. Further research showed that miR-31 overexpression increased the differentiation of Th22 cells by inhibiting BTB domain and CNC homolog 2 (Bach2). These findings indicate that microRNAs may also participate in the regulatory differentiation of Th22 cells.

## The Relationship Between TH22 Cells and Other TH Cells

Both Th22 cells and Th17 cells are T helper cells that are differentiated from naive CD4^+^ T cells (Figure 1). Th17 cells are a subset of CD4^+^ T cells that are independent of Th1 and Th2 that was first identified by Park et al. in their research of autoimmune encephalomyelitis and collagen-induced arthritis (3). Cytokines involved in Th17 cell differentiation include TGF-β, IL-6, IL-1β, IL-21, and IL-23 (4, 32). IL-6 and TGF-β are especially important in this process. TGF-β along with IL-6 initiates Th17 cell differentiation through the RORγt signal transduction pathway (19). Th17 cells can specifically secrete cytokines such as IL-17A, IL-17F, IL-21, IL-22, and TNF-α instead of IL-4 or IFN-γ. This allows Th17 cells to exert their biological effects (4).

Both Th22 cells and Th17 cells are activated to play an immunomodulatory role in some diseases. TNF-α is a cytokine secreted by Th22 cells and Th17 cells. Andersen et al. used anti-TNF-α to treat patients with spondyloarthritis (SpA) for 52 weeks of treatment (33). In this time, the number of Th22 cells and Th17 cells increased continuously, while the expression of IL-23 receptor decreased significantly. Two kinds of cell subsets were positively correlated with the Ankylosing Spondylitis Disease Activity Score and the Bath Ankylosing Spondylitis Activity Index. Increased frequencies of Th22 and Th17 cells in peripheral blood may be related to the activity and duration of the autoimmune thyroid disease (12). Th22 cells and Th17 cells also showed a certain correlation in patients with preeclampsia. Studies have found that the percentage of Th22 cells and Th17 cells in the peripheral blood of patients with preeclampsia was significantly increased and there was a positive correlation between Th22 cells and Th17 cells (34).

In some situations, Th22 cells and Th17 cells restrict each other and behave as opposite immune effects. In immune-mediated skin diseases, such as atopic dermatitis, allergic contact dermatitis, and psoriasis, Th22 cells and Th17 cells jointly activated keratinocytes and initiated non-specific immunity to protect the skin against the invasion of pathogens. In the pre-inflammatory state, the roles of the two types of cells are not exactly the same. Th17 mainly relies on the activation of IFN-γ and IL-17 to enhance the expression of adhesion molecules on keratinocyte, which in turn initiates the T cell-mediated cytotoxicity (35, 36). Th22 cells maintain the integrity of the skin by inducing the proliferation and migration of keratinocytes (37).

Th22 cells and Th17 cells may play roles at different stages of the disease and help to regulate each other. ApoE^−/−^ mice were fed the Western diet in order to induce the atherosclerosis model. It was found that the proportions of both Th22 and Th17 cells increased. Levels of Th17 cells began to decrease at the fourth week and almost declined to the initial levels at the 8th week. Th22 remained at a high level throughout observation. After 12 weeks of feeding and treatment with recombinant mouse IL-22, large plaques appeared in the aorta and the aortic root and increased the levels of Th17 cells, DCs, and pSTAT3. Anti- IL-22 monoclonal neutralizing antibody treatment may have the opposite effect. Further studies have revealed that rIL-22 and ox-LDL stimulation can induce the maturation of bone marrow-derived dendritic cells, which further induces Th17 cell proliferation through the IL-6/STAT3 pathway, thereby aggravating the development of atherosclerosis (38).

The differentiation of Th22 cells is different from that of Th17 cells. RORγt (RORC) is the transcription regulator of Th17 cells, and AHR is the main transcription regulator of Th22 cells. A recent study found that medroxyprogesterone acetate (MPA) could inhibit the expression of IFN-γ, IL-22, IL-17A, and RORC in Th17/Th1 cell clones of peripheral blood, but MPA also significantly increased the expression of AHR, T-bet, and IL-22 in Th22 cell clones (39). TGF-β is an important differentiation factor for Th17 cells, but it exerts an inhibitory effect on Th22 cell differentiation. *In vitro*, activated B cells and naive T cells were co-cultured under Th17 cell culture conditions. It was found that activated B cells could significantly inhibit the production of IL-17 and the expression of RORγt, but were able to stimulate Th22 differentiation and IL-22 production (27).

Th1 cells mainly secrete IL-2, IL-12, IFN-γ, TNF-α, and TNF-β, participate in cellular immunity, activate cytotoxic T lymphocytes and macrophages, mediate organ specific immune response, and eliminate intracellular pathogens. Wolk et al. discovered that IL-22 expression could be increased by inducing Th1 polarization *in vitro* (40). The percentage of Th1 and Th22 cells in untreated immune thrombocytopenia patients was significantly higher than that in healthy controls. IL-22 levels were positively correlated with the proportion of Th1 and Th22 cells. After dexamethasone treatment, the number of Th1 cells and Th22 cells significantly decreased and the level of IL-22 notably declined as well. The polarization of Th1 cells and Th22 cells may contribute to IL-22 expression (41). T-bet is a specific transcription factor for Th1 cell differentiation that mediates the specific expression of IFN-γ in Th1 cells and can inhibit the differentiation of Th2 cells. However, T-bet exerts an inhibitory effect on the differentiation of Th22 cells. Under the condition of Th1 differentiation, 30–50% of Th22 cells can express IFN-γ (24). This indicates that Th22 cells have plasticity to differentiate into Th1 under specific environmental conditions. Th1, Th17, Th22, and Treg cells maintain a balance and jointly regulate the progress of some autoimmune diseases (42). In chronic myelogenous leukemia (CML), Th22, Th17, and Th1 levels were significantly reduced in both the bone marrow and peripheral blood in patients newly diagnosed with CML. These levels were inversely correlated with the percentage of BCR-ABL gene fusion [BCR-ABL (%) IS] (43).

The cytokines secreted by Th2 cells mainly include IL-4, IL-5, IL-6, IL-13, and IL-10. These cytokines can promote the proliferation and differentiation of B cells as well as induce and promote humoral immunity. Cytokines such as IL-6 could promote the differentiation of Th22 cells. In fact, activation of Th2 and Th22 produced differences in disease states. During the acute phase of atopic dermatitis, the bias of Th2 and Th22 cytokines was observed, while Th1 and Th17 cytokines did not show a significant increase (44). Th2 cell activation could be observed in both intrinsic and extrinsic atopic dermatitis (AD). When compared with extrinsic AD, intrinsic AD showed more prominent immune activation, especially on the Th22/Th17 axes (45). In Alopecia areata, Th1 and Th2 cells were activated, but markers of Th17/Th22 cells did not increase significantly (46).

## The Roles of TH22 Cells in Human Viral Diseases

With continuous research, studies have found that Th22 cells play a regulatory role in the occurrence and development of many diseases (Table 1). The cellular and humoral immune responses play an important role in the pathogenesis of viral infectious diseases. In addition to Th1, Th2, Th17, Tfh, and Treg cells, an increasing number of studies have found that Th22, which produces IL-22, is involved in the pathogenesis of multiple viral infectious diseases. Both pathologic and protective roles have been attributed to Th22 in maintaining immunologic homeostasis.

**Table 1:** The role of Th22 cells in human viral diseases.

**Diseases**	**Th22 cells and IL-22**	**Associated molecules**	**Roles**	**References**
AIDS	Th22 cells, IL-22, 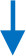 MAIT	CCR5, CCR10, CCL28	Protect mucous membrane	(44–49)
Hepatitis B	Th22 cells, IL-22 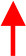	STAT3, IL-22R	Anti-inflammatory or pro-inflammatory, promote the proliferation of liver stem/progenitor cells (LPCs), inhibit hepatocyte apoptosis.	(50–54)
Hepatitis C	Th22 cells, IL-22 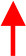	IL-22BP, IL-22R, Notch signaling proteins	Promote the expression of pro-inflammatory factors, reduce the apoptosis of liver cells, activate hepatic stellate cells (HSCs)	(55–64)
HFMD	Th22 cells, IL-22 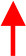	AhR and RORγt	Related to the severity of the disease	(65, 66)
COVID-19	Th22 cells, IL-22 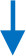 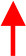	Unclear	Negatively related to the severity of the disease	(67–70)
IAV infection	Th22 cells, IL-22 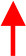	AhR and RORγt, IL-22BP, IL-22Ral	Reduce pulmonary inflammation	(71–75)

*AIDS, Acquired Immune Deficiency Syndrome; HFMD, hand, foot, and mouth disease; COVID-19, coronavirus disease 2019; IAV, influenza A virus; CCR5, chemokine receptor 5; CCR10, chemokine receptor 10; MAIT, mucosal-associated invariant T cells; STAT3, Signal Transducer and Activator of Transcription 3; AhR, aryl hydrocarbon receptor; IL-22BP, IL-22 binding protein; IL-22Ra1, IL-22 receptor a1; 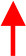, Increased; 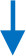, Reduced*.

Studies have shown that Th22 cells play a protective role in the process of HIV infection. Kim et al. found that the expression of Th22 cells in the HIV-infected sigmoid colon mucosa was dramatically absent and could be reversed after prolonged antiretroviral therapy (9). Th22 cells expressed the molecules CCR5 and α4β7 for binding to HIV receptor in circulation. The recombinant IL-22 can resist HIV-induced destruction of epithelial cell integrity. In some HIV-resistant individuals (HIV-exposed uninfected individuals), the number of Th22 cells was significantly higher than that of healthy controls and HIV-infected people. Some protein molecules, including IL-22, were involved in the innate host resistance mechanism (47, 48). In HIV-infected children, Th22 cells and mucosal-associated invariant T cells (MAIT) were significantly reduced. There was a clear correlation after successful antiretroviral therapy (ART). Th22 cells in circulation were significantly increased, suggesting that the proliferation of cTh22 could provide immunological advantages for suppressing HIV-1 infection (49). The expression of Th22 and Th17 cells were decreased in the mucosal tissues of HIV infected patients (50). Th17 cells are incompletely restored at normal frequencies in most HIV-infected individuals on antiretroviral therapy,but Th22 cells complete migration through the CCR10-CCL28 axis, which plays a protective role in the mucosa (51). These findings implied that Th22 cells played more important protective role than that of Th17 cells in HIV infection.

Hepatitis B virus infection is the main cause of liver cirrhosis and liver cancer. Th22 cells in the blood of patients with hepatitis B virus infection were found to be increased, along with significantly higher levels of IL-22 (10, 52, 53). The levels of Th22 cells and IL-22 were related to the severity and prognosis of the disease (10, 54). Injection of IL-22 could promote the expression of pro-inflammatory genes in the liver of HBV transgenic mice instead of directly inhibiting virus replication. Transplanting spleen cells from HBV-immunized mice to HBV transgenic mice and neutralizing IL-22 could reduce liver damage in model mice and significantly inhibited the accumulation of antigen-non-specific inflammatory cells to the liver (53). These results indicated that IL-22 has a pro-inflammatory effect during HBV infection. Other research suggests that IL-22 plays a protective role in liver damage. Radaeva et al. detected the increase of IL-22 and IL-22 receptors in the hepatitis model induced by Concanavalin A (ConA). An IL-22 neutralizing antibody could aggravate liver injury, and overexpression of IL-22 was found to activate STAT3 and reduce the apoptosis of hepatocytes (55). In chronic HBV infected patients and animal models, IL-22 was able to promote the proliferation of liver stem/progenitor cells (LPCs) through activation of the STAT3 pathway (54). These reports indicated that Th22 cells and IL-22 played a protective role in HBV infection.

Chronic hepatitis C (CHC) is another common viral hepatitis. Th22 cells and IL-22 are significantly increased in blood and liver of CHC patients, which play an important role in regulating liver immunity (11, 56, 57). IL-22 was involved in hepatofibrosis (HF) and cirrhosis associated with HCV infection. On one hand, IL-22 can activate the innate immunity of liver, promote the expression of pro-inflammatory factors, promote the proliferation, migration and tissue regeneration of liver cells, and reduce the apoptosis of liver cells (58–60). Hence, it may be an effective target for the treatment of liver fibrosis and HCC. Overexpression of IL-22 binding protein (IL-22BP), which is a competitive inhibitor of IL-22, can aggravate the progression of liver fibrosis and cirrhosis (60). On the other hand, it has been reported that high levels of IL-22 positive cells and IL-22 in intrahepatic and peripheral blood are positively correlated with the progression of liver fibrosis and α- smooth muscle actin (α- SMA) level (61). IL-22 can activate hepatic stellate cells (HSCs) by binding with IL-22R1, which can increase the synthesis of extracellular matrix (ECM) and aggravate HCV associated liver fibrosis (61). IL-22 can promote the proliferation of HSCs *in vitro* and accelerate the progression of liver fibrosis from hepatitis C virus recurrence after orthotopic liver transplantation (HCV-OLT) (62). Notch signaling promotes IL-22 secretion by regulating the expression of AhR. The levels of Th22 cells and Notch signal in peripheral blood of CHC patients were significantly increased. Inhibition of Notch signal could reduce the expression of Th 22 cells, IL-22 and AhR in HCV infected patients (63). The imbalance of Tregs and Th17 cells is a key factor of persistent chronic HCV infection. Th17 mediated immune response could be inhibited when Notch signal is suppressed, and the expression of ROR-γand IL-17/IL-22 was decreased (64). HCV is an RNA virus whose genetic material can be directly integrated into the host genome, which increases the risk of HCC. HCV core protein is critical to drive the transformation of normal hepatocytes into cancer cells. Suppressor of cytokine Signaling 3 (SOCS-3) proteins can cause the dysfunction of IL-22-mediated hepatocyte regeneration, and HCV core protein and SOCS-3 are highly expressed in patients with liver cirrhosis and HCC (58). These findings indicate that Th22 cells and associated molecules play a crucial role in the pathogenesis of HCV infection.

Hand, foot, and mouth disease is an infectious disease caused by enteroviruses. Coxsackievirus A16 (CV-A16) and enterovirus 71 (EV-71) are the most common pathogens that cause this disease. Previous studies found that levels of Th22 cells in the peripheral blood of EV-71 associated severe patients and mild patients were significantly higher than those in healthy controls (65). The levels of IL-22, IL-17A, IL-23, IL-6, TNF-α, AhR, and RORγt were different among mild patients, severe patients, and healthy controls. In convalescent patients, Th22 cells decreased significantly. This research suggests that Th22 cells play an important role in the pathological process of EV-71 infection. IL-22 may have different pathological effects in hand, foot, and mouth disease that is caused by different pathogens. Research has found that hand, foot, and mouth disease patients with encephalitis had higher levels of IL-5, IL-22, and IL-23 (66). In addition, those with EV-71 infection had higher levels of IL-22 than those with CV-A16 infection. These results indicated that Th22 cells and associated molecules were associated with the severity of HFMD caused by CV-A16 or EV-71 infection.

The outbreak of the coronavirus disease 2019 (COVID-19), caused by severe acute respiratory syndrome coronavirus 2 (SARS-CoV-2), is a serious threat to human health. The immunological dynamics in SARS-CoV-2 infected patients has been investigated by many researchers. Daniela Fenoglio *et al*. analyzed Th subsets in peripheral blood of 13 patients with severe COVID-19 and 10 healthy controls, and found that the frequencies of Th1 and Th17-1 cell were reduced in COVID-19 patients (67). Another study observed a significantly lower lymphocyte count in COVID-19 patients compared to healthy controls. They found that the percentages of Th1, Th1/Th17, and TFH cells were significantly reduced in both non-ICU hospitalized and ICU hospitalized patients compared to healthy controls. There is some controversy about Th22 cells (68). Daniela Fenoglio *et al*. found that there was no significant difference about Th22 between COVID-19 patients and controls (67). However, Juan Francisco Gutie'rrez-Bautista *et al*. found that Th22 showed the opposite change. Th22 was slightly elevated in non-ICU hospitalized patients and asymptomatic recovered donors, but significantly reduced in ICU hospitalized patients compared to healthy controls (68). This may be related to the excessive consumption of Th22 in severe patients. In addition, functional assays revealed that the ability to produce IL-22 of peripheral blood mononuclear cell (PBMCs) from critically ill patients was significantly lower than that of health controls (69). However, another study suggested that IL-22 was highly expressed in the infected human bronchial epithelial cell line (16HBE) (70). This suggested IL-22 showed different distribution in peripheral blood and tissues.

Influenza is another common disease that endangers human health. IL-22, the main effector of Th22, plays a crucial role in influenza A virus (IAV) infection. It has been indicated that a higher level of IL-22 expression was detected in the lung tissue during the early stages of IAV infection (71, 72). RORγt and aryl hydrocarbon receptor was crucial in IL-22 synthesis after IAV infection (71). During IAV infection, IL-22 plays protective role in lung injuries.but,IL-22 does not appear to affect pulmonary pathogenesis during lethal IAV infection (71). Interestingly, when human peripheral blood mononuclear leukocytes (PBML) exposed to vaccines against influenza virus, the level of IL-6, IL-1β, TNF-α and IL-22 obviously increased (73). This further suggested that the influenza virus can activate the expression of IL-22. After influenza virus infection, IL-22 may reduce pulmonary inflammation via IL-22Ra1 or the IL-22/ IL-22BP axis (74, 75).

Notably, in addition to Th22 cells, IL-22 can also be derived from Th17 cells, natural killer T cells, γδT cells, and type 3 ILCs. The pathogenesis of each disease is intricate. Various cell subsets regulate each other and collectively participate in the process of the disease. The mechanism of Th22 cells in disease still needs more complete research.

## Conclusion

The exploration of T cells has made rapid progress over the last 20 years, and newly discovered T cell subsets show a variety of differentiation characteristics. Various cell subsets are able to regulate each other in order to keep the body in a delicate and complex balance. The discovery of Th22 cell subsets further enriches the complex system of immune regulation gridding and illustrates the high plasticity of naive T cells. IL-22 is the main effector of Th22 cells, and AhR is the differentiation transcription factor of Th22 cells that can be promoted by IL-6, TNF-α, AhR agonists, and TGF-β receptor inhibitors. TGF-β and IL-10 can inhibit the differentiation of Th22 cells. The function of Th22 cells extends from the initial involvement in skin inflammation and wound healing to the regulation of pathological processes such as autoimmune diseases, infectious diseases, tumors, hematological diseases, and kidney diseases. Circulating Th22 cells can migrate to pathological tissues through chemokine receptors, express cytokines, and bind to the corresponding receptors. This allows Th22 cells to elicit biological effects. In some diseases, Th22 cells and their effector molecules play a protective role; however, in other diseases, Th22 cells can aggravate the disease progression. The different roles of Th22 cells and associated molecules are closely associated with the types of viral diseases and duration of viral infection. Although the study of Th22 cell has been extended to various systems, the understanding of this precise regulatory mechanism is still very limited. In addition, the regulation of Th22 cell differentiation and downstream pathways are still not completely clear. The relationship with Th17, Th9, Th1, and other cells is not completely understood, along with whether it is involved in the regulation of humoral immunity. Further research should be done to examine the differentiation and regulation mechanism of Th22 cells, explore their interaction with other immune cells, and analyze their mechanism in various diseases. These will help to provide new targets and strategies for diagnosis and treatment of many diseases.

## Author Contributions

DC and QH designed the study and revised the manuscript. JG, DC, and HZ drafted the manuscript. JG and YL drew the figure and Table. All authors have read and approved the final version of the manuscript.

## Conflict of Interest

The authors declare that the research was conducted in the absence of any commercial or financial relationships that could be construed as a potential conflict of interest.

## Publisher's Note

All claims expressed in this article are solely those of the authors and do not necessarily represent those of their affiliated organizations, or those of the publisher, the editors and the reviewers. Any product that may be evaluated in this article, or claim that may be made by its manufacturer, is not guaranteed or endorsed by the publisher.
